# Supplementary material for: Impact of maternal antibodies and microbiota development on the immunogenicity of oral rotavirus vaccine in African, Indian, and European infants
Source: Nat Commun. 2021 Dec 15;12:7288. doi: 10.1038/s41467-021-27074-1 (PMC8674366; doi:10.1038/s41467-021-27074-1)
Supplement: Supplementary file 1 — Supplementary Information [file 41467_2021_27074_MOESM1_ESM.pdf]

Supplementary Table 1. Cofactors Associated with Neonatal Rotavirus Infection in India.

variable	N, total (exposed)	neo+	neo–	RR (95% CI)	p value
infant characteristics at baseline					
female, n/N (%)	304 (166)	85/166 (51.2%)	64/138 (46.4%)	1.09 (0.88–1.29)	0.402
**delivery by caesarean section, n/N (%)**	**304 (166)**	**46/166 (27.7%)**	**23/138 (16.7%)**	**1.31 (1.05–1.53)**	**0.023**
**delivered in tertiary care facility, n/N (%)**	**304 (166)**	**98/166 (59.0%)**	**30/138 (21.7%)**	**1.98 (1.72–2.19)**	**<0.001**
exclusively breastfed, n/N (%)	304 (166)	145/166 (87.3%)	118/138 (85.5%)	1.08 (0.76–1.37)	0.640
age at first ORV dose (days), mean (sd)	304 (166)	42.6 (1.5)	42.6 (1.1)	0.98 (0.90–1.06)	0.680
birthweight (kg), mean (sd)	304 (166)	2.9 (0.4)	3.0 (0.4)	0.94 (0.69–1.17)	0.602
height-for-age *Z* score (week of life 6), mean (sd)	303 (166)	–0.5 (1.1)	–0.5 (1.1)	1.01 (0.91–1.10)	0.889
exposed to antibiotics during study, n/N (%)	304 (166)	48/166 (28.9%)	36/138 (26.1%)	1.07 (0.83–1.29)	0.583
polio vaccines: bOPV only	304 (166)	57/166 (34.3%)	37/138 (26.8%)	ref	*LRT: p = 0.291*
IPV	304 (166)	47/166 (28.3%)	51/138 (37.0%)	0.79 (0.56–1.02)	0.079
mixed tOPV/bOPV	304 (166)	29/166 (17.5%)	27/138 (19.6%)	0.85 (0.58–1.12)	0.290
tOPV only	304 (166)	33/166 (19.9%)	23/138 (16.7%)	0.97 (0.70–1.22)	0.836
maternal characteristics at baseline					
age (years), mean (sd)	304 (166)	23.5 (3.7)	23.4 (3.7)	1.00 (0.98–1.03)	0.821
weight (kg), mean (sd)	304 (166)	61.0 (10.7)	61.2 (10.4)	1.00 (0.99–1.01)	0.840
parity, mean (sd)	304 (166)	0.8 (0.9)	1.0 (0.9)	0.90 (0.78–1.01)	0.086
completed years of education, mean (sd)	304 (166)	10.0 (3.7)	9.8 (3.7)	1.01 (0.98–1.04)	0.633
household characteristics					
**house made of permanent materials, n/N (%)**	**304 (166)**	**120/166 (72.3%)**	**78/138 (56.5%)**	**1.40 (1.13–1.64)**	**0.004**
access to treated water, n/N (%)	287 (157)	78/157 (49.7%)	53/130 (40.8%)	1.18 (0.95–1.39)	0.132
access to sanitation facility (e.g., latrine), n/N (%)	304 (166)	77/166 (46.4%)	59/138 (42.8%)	1.07 (0.86–1.27)	0.526
kitchen in home, n/N (%)	304 (166)	118/166 (71.1%)	89/138 (64.5%)	1.15 (0.91–1.38)	0.220
**refrigerator in home, n/N (%)**	**304 (166)**	**103/166 (62.0%)**	**69/138 (50.0%)**	**1.25 (1.02–1.47)**	**0.035**
>4 people per room, n/N (%)	304 (166)	79/166 (47.6%)	68/138 (49.3%)	0.97 (0.77–1.17)	0.770
highest income in household (rupees), mean (sd)*	304 (166)	8116.2 (7431.4–8864.1)	7496.9 (6759.7–8314.4)	1.10 (0.93–1.27)	0.247
inflammatory biomarkers					
α1AT (μg/ml, week of life 6), GMC (95% CI)*	299 (163)	713.5 (639.0–796.8)	755.0 (666.3–855.6)	0.95 (0.81–1.09)	0.501
MPO (ng/ml, week of life 6), GMC (95% CI)*	299 (163)	6364.6 (5042.7–8033.1)	6481.4 (5003.6–8395.8)	1.00 (0.93–1.06)	0.917
α1AG (μg/ml, week of life 6), GMC (95% CI)*	303 (165)	713.8 (687.0–741.8)	733.3 (703.0–764.8)	0.80 (0.44–1.21)	0.351
maternal antibodies					
maternal RV-IgA (IU/ml), GMC (95% CI)*	304 (166)	131.1 (113.2–151.7)	137.9 (114.9–165.5)	0.98 (0.87–1.08)	0.662
breastmilk RV-IgA (IU/ml), GMC (95% CI)*	300 (163)	23.6 (19.7–28.4)	27.6 (22.2–34.3)	0.95 (0.87–1.04)	0.279
maternal RV-IgG (IU/ml), GMC (95% CI)*	304 (166)	9642.1 (8304.8–11194.6)	10475.8 (8834.8–12421.7)	0.96 (0.86–1.06)	0.467
cord blood RV-IgG (IU/ml), GMC (95% CI)*	304 (166)	13497.1 (11761.3–15489.1)	14278.5 (11873.4–17170.9)	0.97 (0.87–1.08)	0.622
**infant RV-IgG pre-ORV (IU/ml), GMC (95% CI)***	**304 (166)**	**9430.1 (8298.8–10715.7)**	**5747.1 (4817.1–6856.6)**	**1.24 (1.14–1.34)**	**<0.001**
gut microbiota diversity (genus level)					
Shannon index (week of life 1), mean (sd)	286 (155)	1.3 (0.4)	1.2 (0.4)	1.16 (0.88–1.40)	0.254
Shannon index (week of life 4), mean (sd)	284 (156)	1.2 (0.4)	1.2 (0.4)	1.18 (0.93–1.39)	0.162
Shannon index (week of life 6), mean (sd)	286 (154)	1.3 (0.4)	1.3 (0.4)	1.01 (0.74–1.27)	0.948

Neonatal infection (neo+) was defined by detection of rotavirus shedding in week of life 1 or baseline seropositivity. Logistic regression was used to explore the relationship between each covariate and neonatal infection.

α1AG, α1 acid glycoprotein; α1AT, α1-antitrypsin; bOPV, bivalent oral poliovirus vaccine; GMC, geometric mean concentration; IPV, inactivated poliovirus vaccine; LRT, likelihood ratio test for fit of model with vs without polio vaccine schedule; MPO, myeloperoxidase; RR, relative risk; RV, rotavirus; tOPV, trivalent oral poliovirus vaccine; * log-transformed to approximate normality in statistical models.

Supplementary Table 2. Longitudinal Models of Taxon Abundance in Relation to Oral Rotavirus Vaccine Response.

	IND	MLW	UK	IND (neo+)	IND (neo–)
Outcome 1: week of life					
N infants	305	96	51	166	138
N samples (weeks of life 1, 4, 6*, and 10*)	1140	263	197	625	511
N genera tested	25	42	25	26	24
+ correlation (FDR p <0.05)	7	15	4	8	8
– correlation (FDR p <0.05)	6	11	11	7	6
Outcome 2: seroconversion					
N infants	305	96	51	166	138
N samples (weeks of life 1, 4, 6*, and 10*)	1140	263	197	625	511
N genera tested	25	42	25	26	24
+ correlation (FDR p <0.05)	0	0	0	0	0
– correlation (FDR p <0.05)	0	0	0	0	0
Outcome 3: post-vaccination RV-IgA					
N infants	305	96	51	166	138
N samples (weeks of life 1, 4, 6*, and 10*)	1140	263	197	625	511
N genera tested	25	42	25	26	24
+ correlation (FDR p <0.05)	0	0	0	0	0
– correlation (FDR p <0.05)	0	0	0	1†	0
Outcome 4: shedding after dose 1					
N infants	305	89	–	164	138
N samples (weeks of life 1, 4, and 6)	859	189	–	459	391
N genera tested	23	44	–	24	22
+ correlation (FDR p <0.05)	0	0	–	0	0
– correlation (FDR p <0.05)	0	1§	–	0	0

Discrepancies in taxon abundance were assessed using zero-inflated negative binomial models (or negative binomial models if the taxon was observed in >95% of samples) that included study ID as a random effect. Taxa with a prevalence of ≥20% in each cohort were assessed. Age was included as a covariate in analyses of oral rotavirus vaccine outcome.

FDR, false discovery rate; IND, India; MLW, Malawi; neo+, infected with rotavirus neonatally (defined by detection of rotavirus shedding in week of life 1 or baseline seropositivity); neo-, uninfected with rotavirus neonatally; RV, rotavirus; * +2 weeks in UK due to later vaccination schedule; † *Lactobacillus* negatively correlated with RV-IgA (prevalence = 62.7%; coefficient = -0.181; FDR p = 0.036); § *Sutterella* negatively correlated with shedding after dose 1 (prevalence = 43.9%; coefficient = -1.417; FDR p = 0.010).

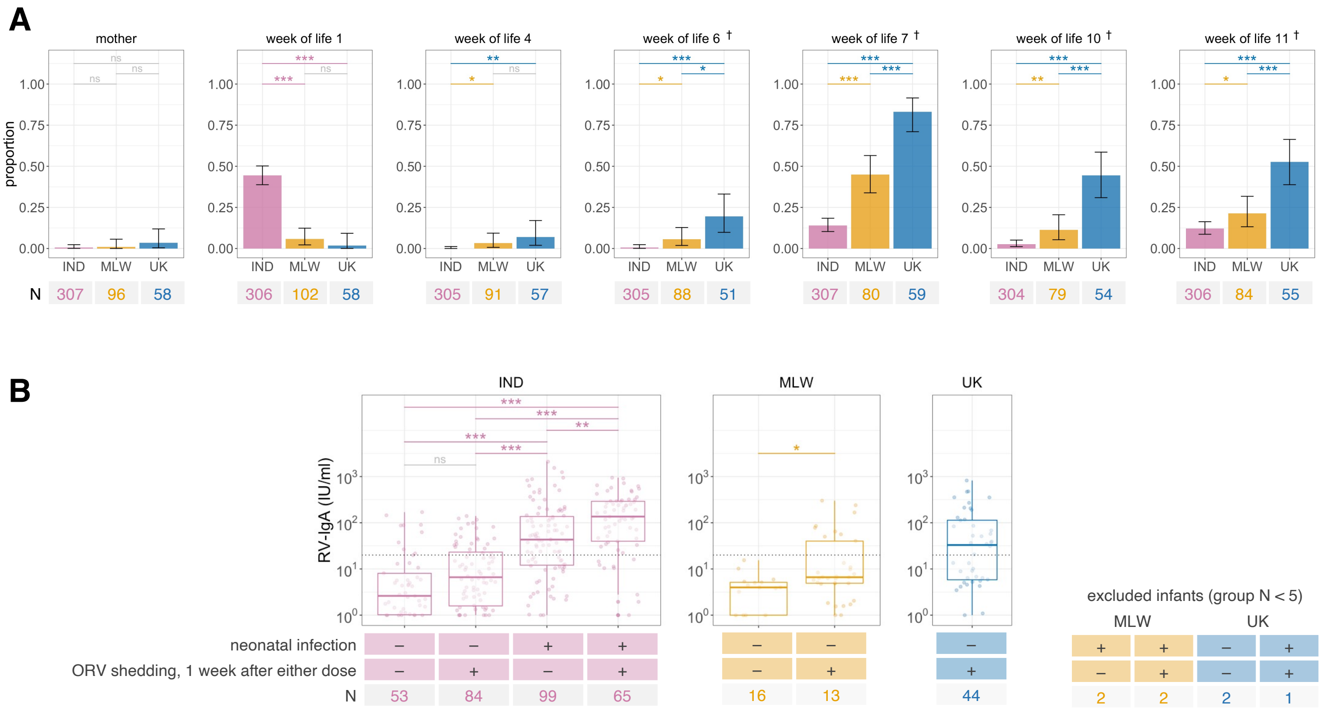

Supplementary Fig. 1: Longitudinal Analysis of Rotavirus Shedding and Overall Immunogenicity. (A) Rotavirus shedding across all timepoints, as determined via quantitative PCR targeting the *VP6* gene of group A rotaviruses. Error bars represent Clopper–Pearson 95% confidence intervals. Groups were compared by two-sided Fisher's exact tests with FDR correction. (B) Cumulative immunogenicity of neonatal infection and ORV shedding. Post-ORV shedding was detected via quantitative PCR targeting the Rotarix *NSP2* gene. Neonatal infection was defined as the detection of wild-type rotavirus shedding in week of life 1 or baseline seropositivity (RV-IgA ≥20 IU/ml before dose 1). Log-transformed RV-IgA concentrations were compared by ANOVA with post-hoc Tukey tests. Infants with incomplete shedding or RV-IgA data were excluded. See **Fig. 1** legend for box plot parameters. The dotted lines at 20 IU/ml indicate the standard cut-off for RV-IgA seropositivity. IND, India; MLW, Malawi; ns, not significant; RV, rotavirus; †, +2 weeks in UK due to later vaccination schedule; *, p <0.05; **, p<0.005; ***, p <0.0005.

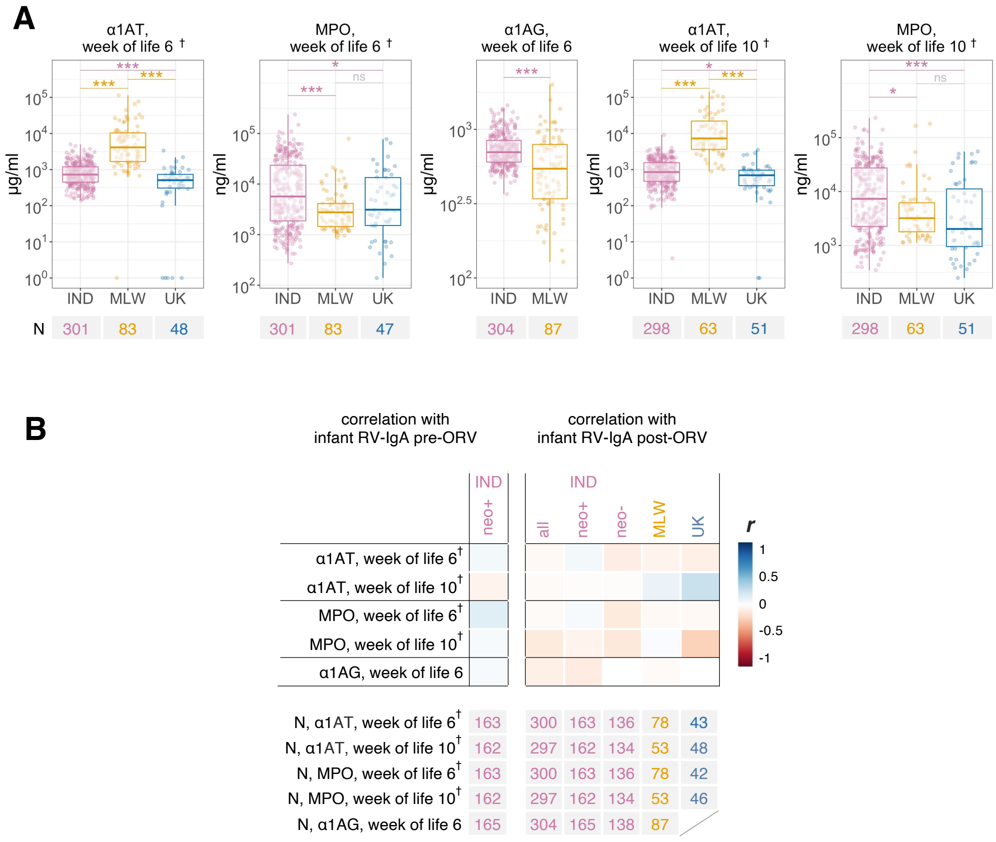

Supplementary Fig. 2: Association Between Environmental Enteric Dysfunction and Oral Rotavirus Vaccine Response. (A) EED markers by cohort. Groups were compared by ANOVAs with post-hoc Tukey tests. α1AG assays were not performed for the UK owing to the limited pre-vaccination sample volumes available for this population. See **Fig. 1** legend for box plot parameters. (B) Association between EED markers and RV-IgA formation. Log-transformed concentrations were compared using Pearson's correlation coefficient (r) with two-sided hypothesis testing. Infant samples for RV-IgA measurement were collected before dose 1 (week of life 6 in India/Malawi; week of life 8 in the UK) and 4 weeks after dose 2 (week of life 14 in India/Malawi; week of life 16 in the UK). α1AT, α1-antitrypsin; α1AG, α1 acid glycoprotein; IND, India; MLW, Malawi; MPO, myeloperoxidase; neo+, infected with rotavirus neonatally (defined by detection of rotavirus shedding in week of life 1 or baseline seropositivity); neo-, uninfected with rotavirus neonatally; ns, not significant; ORV, oral rotavirus vaccine; RV, rotavirus; †, +2 weeks in UK due to later vaccination schedule; *, p <0.05; ***, p <0.0005.

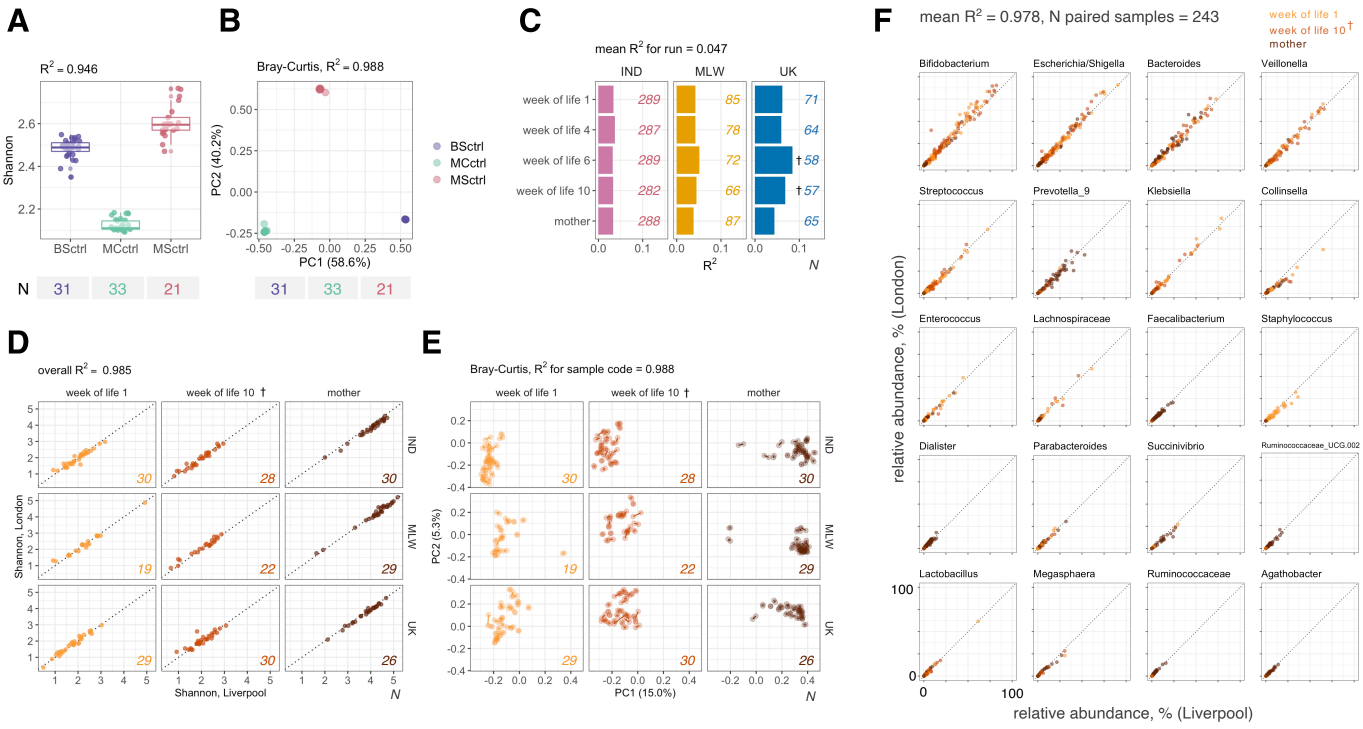

Supplementary Fig. 3: Replicability of Microbiota Sequencing Across Runs and Facilities. Positive controls including an infant stool (BSctrl), maternal stool (MSctrl), and a mock bacterial community (MCctrl) were included in every 96-well sequencing plate, with up to up to four plates per run. (A and B) Run-to-run variation in control composition. Alpha diversity (A) and beta diversity (B) for positive controls at ribosomal sequence variant level were highly replicable from run to run, with sample type accounting for the majority of variation among controls based on linear regression (for Shannon index) and PERMANOVA of unweighted Bray–Curtis distances (for beta diversity). See **Fig. 1** legend for box plot parameters. (C) Proportion of variation associated with run for each sample group. R^2 and statistical significance were determined separately for each sample group by PERMANOVA of unweighted Bray–Curtis distances. Sample counts are indicated in italics. (D–F) 10% validation results. For 90 infant samples collected in week of life 1, 90 infant samples collected at the time of dose 2 (week of life 10 in India/Malawi; week of life 12 in the UK), and 90 maternal samples, we validated the microbiota pipeline at a separate sequencing facility in London. Validation samples were evenly distributed across the study sites (30 per site per sample group) and were randomised across three sequencing plates. PCR, quantification, and pooling all involved the same methods and reagents (ordered separately), although sequencing was performed via Illumina MiSeq rather than HiSeq (used for the majority of runs in Liverpool). Sample pairs were retained in the analysis of both technical replicates had ≥25,000 sequences after quality filtering (243/270 [90%]). Microbiota composition was highly replicable based on alpha diversity (D), beta diversity (E), and relative abundance of major genera (F), with sample ID accounting for >97% of variation based on linear regression or PERMANOVA. Shannon index and unweighted Bray–Curtis distances were calculated at ribosomal sequence variant level. Sample counts are indicated in italics. In (E), technical replicates are linked by a line, although often the overlap is so precise that lines are indistinguishable. IND, India; MLW, Malawi; PC, principal coordinate; †, +2 weeks in UK due to later vaccination schedule.

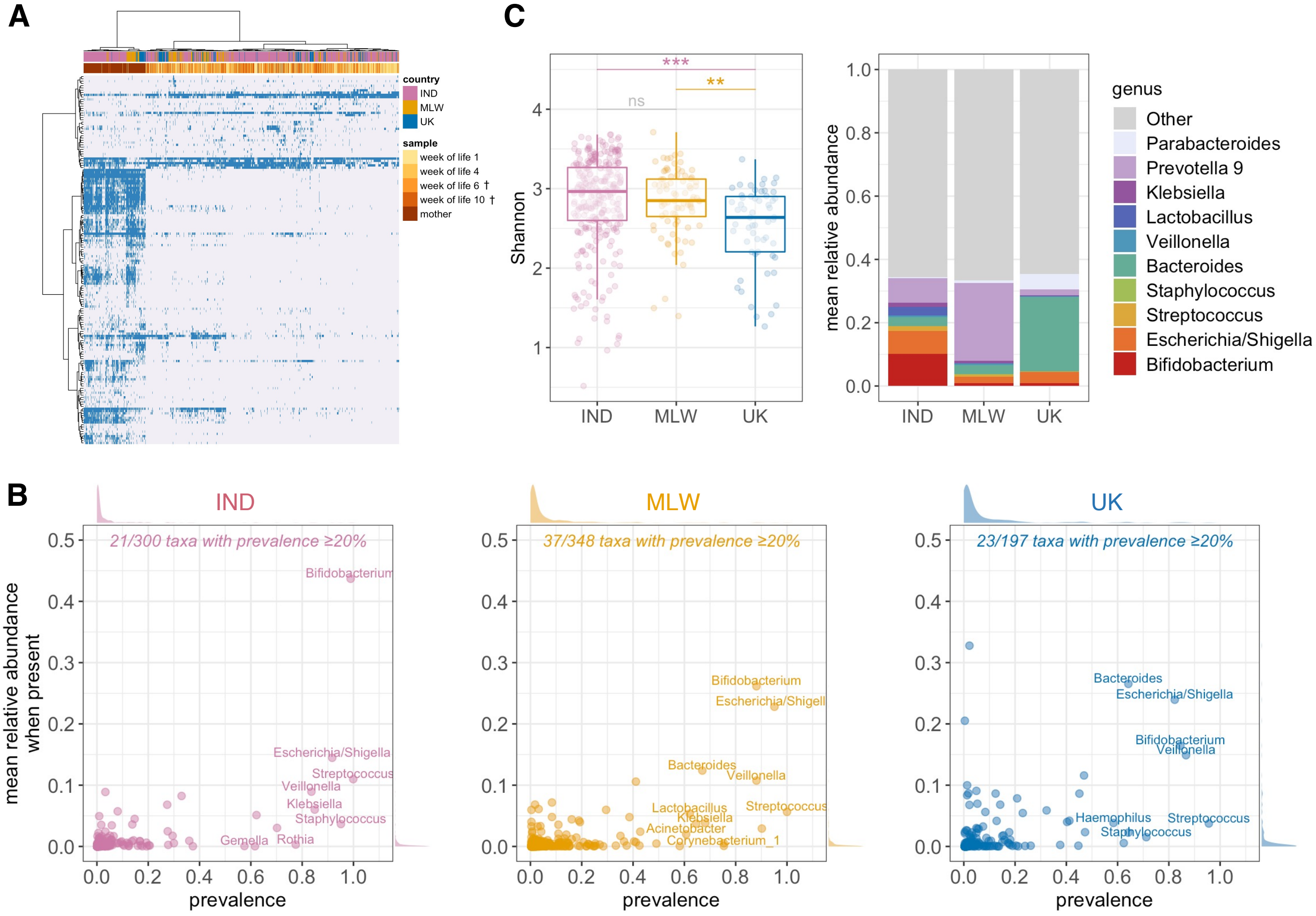

Supplementary Fig. 4: Genus Profiles of Infant and Maternal Samples. (A) Hierarchical clustering of stool samples (columns; n = 2,086) based on presence/absence of common genera (rows). Genera detected in 1% of samples were included (n = 162). Rows and columns were clustered using Ward's minimum variance hierarchical clustering method. (B) Genus abundance profile of infant samples. The infant gut microbiota in each cohort was dominated by a small number of taxa with high prevalence and abundance. All infant samples from each cohort were included in the prevalence and abundance calculations (n = 1,147, 285, and 226 for India, Malawi, and the UK, respectively). Margins display density plots. (C) Geographic discrepancies in maternal microbiota composition, including comparisons of genus-level Shannon index (left panel) and genus composition (right panel). Shannon index was compared using ANOVA with post-hoc Tukey tests. Genera are included if they were abundant among infant samples in at least one cohort (see **Fig. 3C** for details). See **Fig. 1** legend for box plot parameters. IND, India; MLW, Malawi; ns, not significant; †, +2 weeks in UK due to later vaccination schedule; **, p <0.005; ***, p <0.0005.

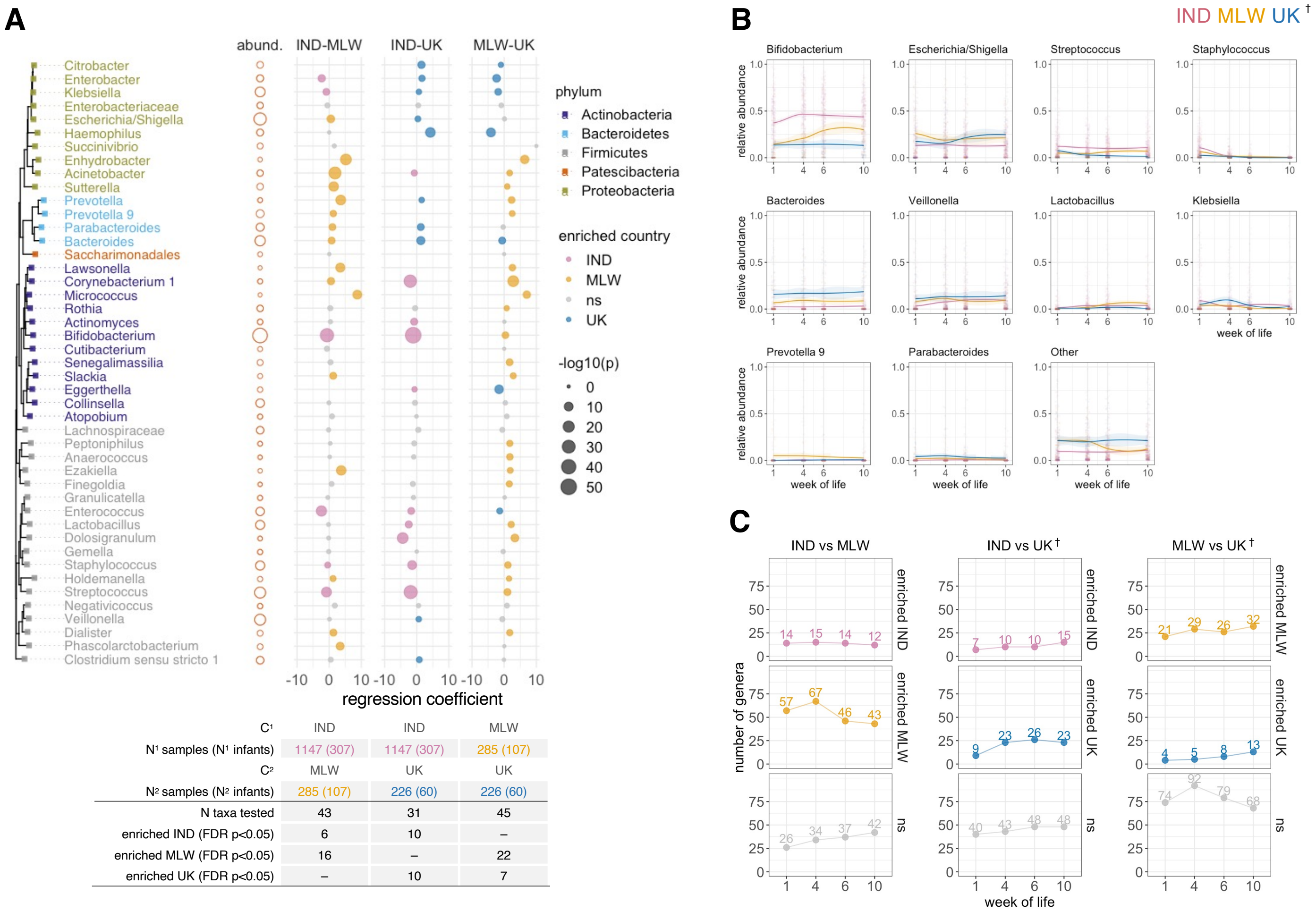

C¹	IND	IND	MLW
N¹ samples (N¹ infants)	1147 (307)	1147 (307)	285 (107)
C²	MLW	UK	UK
N² samples (N² infants)	285 (107)	226 (60)	226 (60)
N taxa tested	43	31	45
enriched IND (FDR p<0.05)	6	10	–
enriched MLW (FDR p<0.05)	16	–	22
enriched UK (FDR p<0.05)	–	10	7

Supplementary Fig. 5: Discriminant Genera by Country. (A) Longitudinal models of genus abundance by country. Discriminant genera were identified using mixed-effects zero-inflated negative binomial models with week of life as a covariate and study ID as a random effect. Genera were included if present in at least 20% of samples from at least one country being compared. Regression coefficients are displayed with points scaled by p value. The phylogeny represents a neighbour-joining tree based on JC69 distances. The most abundant ribosomal sequence variant served as a reference sequence for each genus. Circles to the right of the tree are scaled by mean relative abundance across infant samples (following arcsine square root transformation). (B) Longitudinal relative abundance plots for major genera by country. Genera were included if they had a mean relative abundance of ≥5% in at least one country at one or more timepoints. Lines show local weighted regression (loess) fits with 95% confidence intervals. (C) Cross-sectional comparisons of genus abundance by country. Discriminant genera were identified based on two-sided Fisher's exact test (differences in prevalence) and Aldex2 with two-sided Wilcoxon rank sum test (differences in abundance). The number of genera with an FDR-adjusted p value of <0.05 based on either method is highlighted for each pairwise cross-sectional comparison. See **Supplementary Data 4** for full details of discriminant taxa. C, country; FDR, false discovery rate; IND, India; MLW, Malawi; ns, not significant; †, +2 weeks samples collected at weeks of life 6 and 10 in UK due to later vaccination schedule.

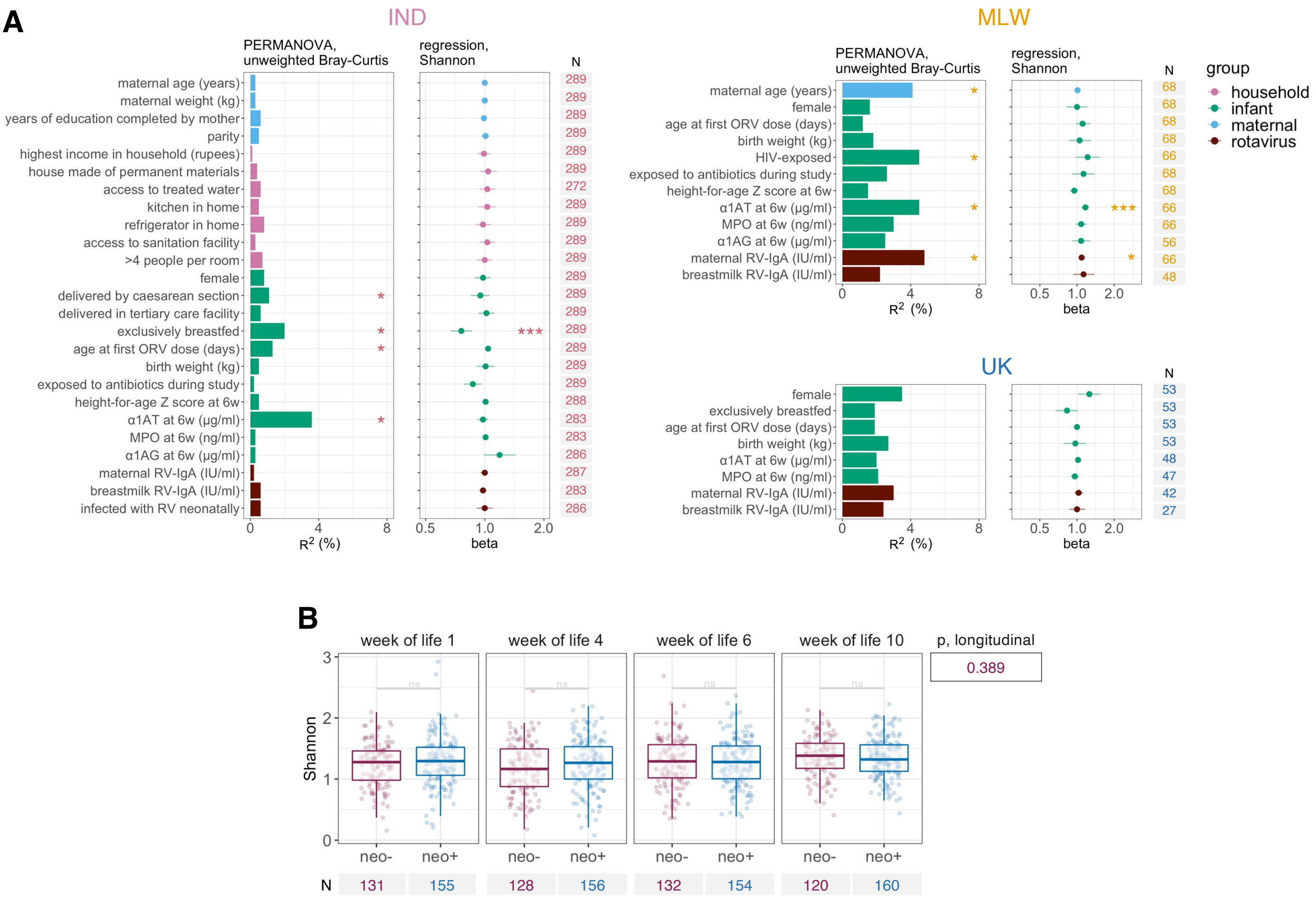

Supplementary Fig. 6: Cofactors Associated with Microbiota Composition. (A) Factors associated with alpha and beta diversity at the time of the first dose of oral rotavirus vaccine (week of life 6 in India/Malawi; week of life 8 in the UK). The left panel, presenting data for Indian infants (n = 289), contains the full list of variables included in this exploratory analyses (with the exception of HIV exposure status, which was also explored for Malawian infants). For analyses of infants from Malawi and the UK (right panels; n = 68 and 53, respectively), variables were excluded if they were not measured or exhibited limited variability (n<10 in either group). PERMANOVA was performed using genus-level unweighted Bray–Curtis distances. Shannon index was calculated at genus level and assessed as an outcome variable via linear regression. (B) Association between neonatal rotavirus infection and Shannon index in Indian infants. Cross-sectional comparisons were performed using logistic regression. The longitudinal comparison was performed using a mixed-effects model including week of life as a covariate and study ID as a random effect. See **Fig. 1** legend for box plot parameters. α1AT, α1-antitrypsin; α1AG, α1 acid glycoprotein; IND, India; MLW, Malawi; MPO, myeloperoxidase; neo+, infected with rotavirus neonatally (defined by detection of rotavirus shedding in week of life 1 or baseline seropositivity); neo-, uninfected with rotavirus neonatally; ORV, oral rotavirus vaccine; RV, rotavirus; * FDR p <0.05; *** FDR p <0.0005.

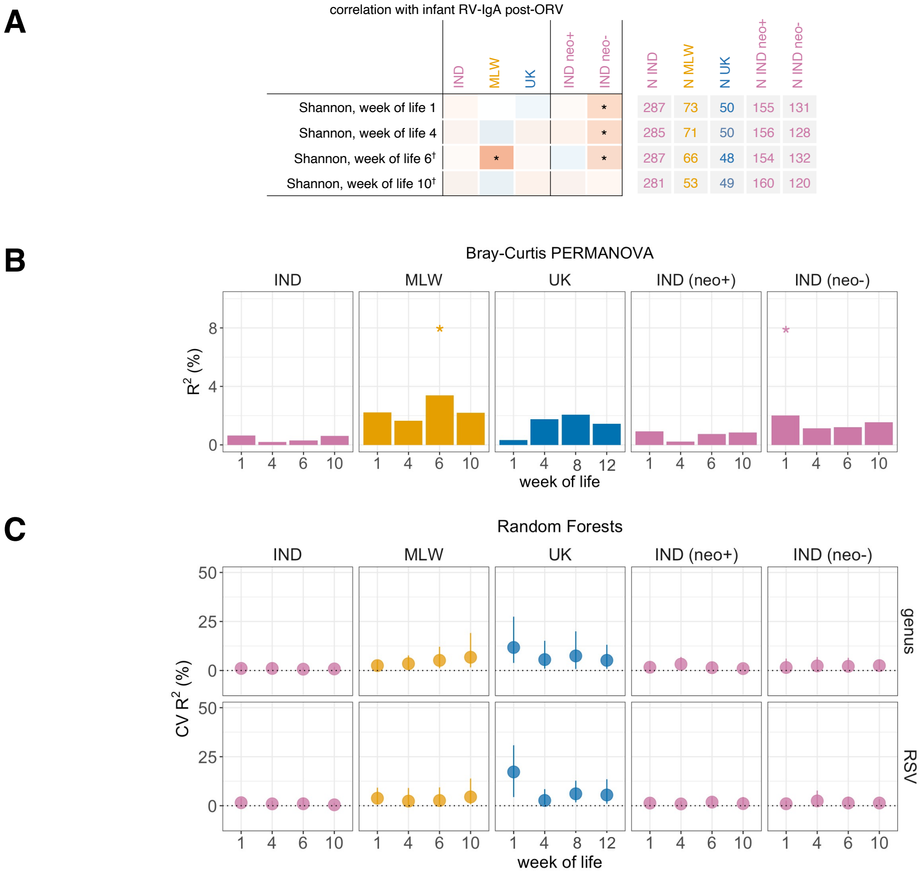

N per RF iteration	287	285	287	281	73	71	66	53	50	50	48	49	155	156	154	160	131	128	132	120
N genera included	44	50	55	61	104	106	94	110	41	65	66	70	46	53	55	60	39	48	56	63
N correlated with RV-IgA (Aldex2)	0	0	0	0	0	0	0	0	0	0	0	0	0	0	0	0	0	0	0	0
N RSVs included	122	130	145	170	249	300	270	300	150	183	189	210	136	158	159	164	136	136	158	181
N correlated with RV-IgA (Aldex2)	0	0	0	0	0	0	0	0	0	0	0	0	0	0	0	0	0	0	0	0

Supplementary Fig. 7: Association between Microbiota Development and Post-Vaccination Rotavirus-Specific IgA Concentration. (A) Correlation between microbiota diversity and RV-IgA. Shannon index was calculated at genus level and compared with log-transformed RV-IgA values using Pearson's correlation coefficient (*r*) with two-sided hypothesis testing. (B) Proportion of variation in microbiota composition associated with RV-IgA. R^2 and statistical significance were determined by PERMANOVA using genus-level unweighted Bray–Curtis distances. (C) Cross-validation accuracy of Random Forests for prediction of post-vaccination RV-IgA. Models were fitted at genus and ribosomal sequence variant level. Median out-of-bag R^2 and interquartile range are displayed for predicted vs observed RV-IgA across 20 iterations of 5-fold cross-validation. Correlations between log-ratio transformed taxon abundance counts and RV-IgA were determined via Aldex2 with two-sided Spearman's rank test. We report the number of taxa with FDR-adjusted p values of <0.05. CV, cross-validation; IND, India; MLW, Malawi; neo+, infected with rotavirus neonatally (defined by detection of rotavirus shedding in week of life 1 or baseline seropositivity); neo-, uninfected with rotavirus neonatally; ORV, oral rotavirus vaccine; RF, Random Forest; RV, rotavirus; RSV, ribosomal sequence variant; * p <0.05.

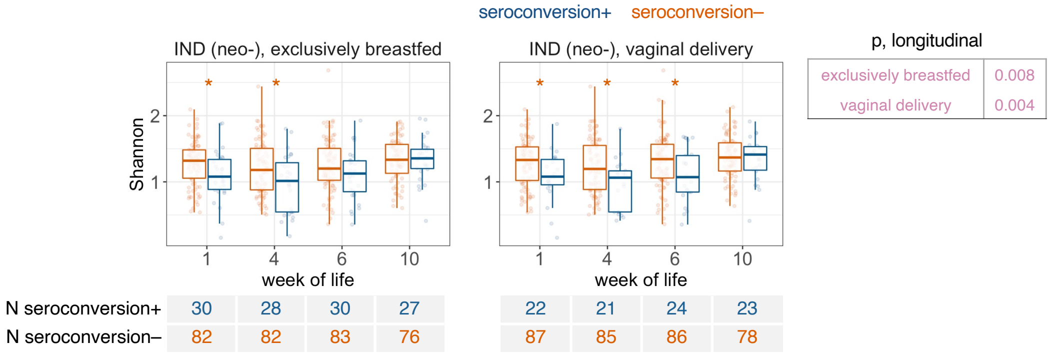

Supplementary Fig. 8. Sensitivity Analysis for Association Between Microbiota Diversity and Oral Rotavirus Vaccine Seroconversion. Infants were included if they were exclusively breastfed (left panel) or born by caesarean section (right panel) to exclude the potential confounding effect of breastfeeding status and mode of delivery. Shannon index was calculated at genus level. Cross-sectional comparisons were performed using logistic regression. Longitudinal comparisons were performed using mixed-effects models including week of life as a covariate and study ID as a random effect**.** See **Fig. 1** legend for box plot parameters. IND, India; neo-, uninfected with rotavirus neonatally; * p <0.05.

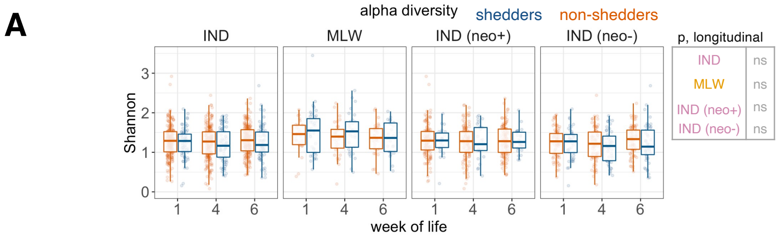

N shedders	73	77	76	39	32	35	26	28	26	46	48	49
N non-shedders	214	208	211	29	32	32	127	126	126	85	80	83

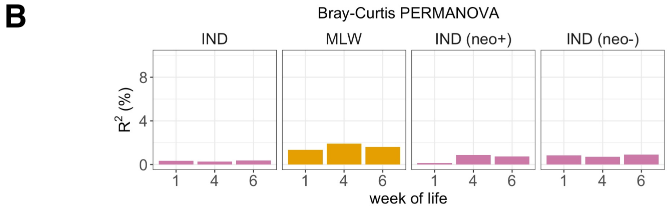

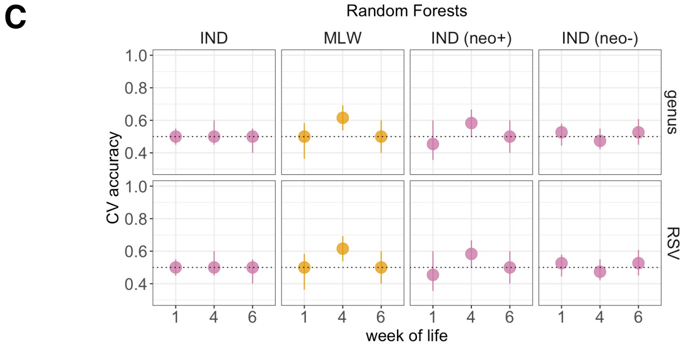

N shedders	73	77	76	39	32	35	26	28	26	46	48	49
N non-shedders	214	208	21	29	32	32	127	126	126	85	80	83
N per RF iteration	100	100	100	58	64	64	52	56	52	92	96	98
N genera included	50	58	65	139	134	120	52	64	67	47	57	64
N differential genera	0	0	0	0	0	0	0	0	0	0	0	0
N RSVs included	164	178	202	459	505	420	182	209	205	161	170	204
N differential RSVs	0	0	0	0	0	0	0	0	0	0	0	0

Supplementary Fig. 9. Association between Microbiota Development and Dose 1 Oral Rotavirus Vaccine Shedding. See **Fig. 4** legend for details; the same analyses of (A) alpha diversity, (B) beta diversity, and (C) Random Forests cross-validation accuracy are presented here with shedding 1 week after the first dose of oral rotavirus vaccine as outcome. Comparisons were not performed for the UK as only 5 out of 60 infants (8.3%) in this cohort were non-shedders. See **Fig. 1** legend for box plot parameters. CV, cross-validation; IND, India; MLW, Malawi; neo+, infected with rotavirus neonatally (defined by detection of rotavirus shedding in week of life 1 or baseline seropositivity); neo-, uninfected with rotavirus neonatally; RF, Random Forest; RSV, ribosomal sequence variant.
