# Supplementary material for: Impact of maternal antibodies and microbiota development on the immunogenicity of oral rotavirus vaccine in African, Indian, and European infants
Source: Nat Commun. 2021 Dec 15;12:7288. doi: 10.1038/s41467-021-27074-1 (PMC8674366; doi:10.1038/s41467-021-27074-1)
Supplement: Supplementary file 3 — Description of Additional Supplementary Files [file 41467_2021_27074_MOESM3_ESM.pdf]

Description of Additional Supplementary Files

File Name: Supplementary Data 1

Description: **Cofactors Associated with Oral Rotavirus Vaccine Shedding After Dose 1.** Logistic regression was used to explore the relationship between each covariate and ORV shedding 1 week after dose 1. Variables with a univariate p value of <0.05 were explored in multivariate models. Measurements obtained after dose 1 (i.e. after week of life 6) were excluded given that the aim was to identify predictors of ORV shedding rather than shedding-induced changes. α1AG, α1 acid glycoprotein; α1AT, α1–antitrypsin; bOPV, bivalent oral poliovirus vaccine; GMC, geometric mean concentration; IND, India; IPV, inactivated poliovirus vaccine; LRT, likelihood ratio test for fit of model with vs without polio vaccine schedule; MLW, Malawi; MPO, myeloperoxidase; neo+, infected with rotavirus neonatally (defined by detection of rotavirus shedding in week of life 1 or baseline seropositivity); neo-, uninfected with rotavirus neonatally; ORV, oral rotavirus vaccine; RR, relative risk; RV, rotavirus; tOPV, trivalent oral poliovirus vaccine; * log–transformed to approximate normality in statistical models; † excluded to minimise multicollinearity.

File Name: Supplementary Data 2

Description: **Cofactors Associated with Oral Rotavirus Vaccine Seroconversion.** Logistic regression was used to explore the relationship between each covariate and seroconversion. Variables with a univariate p value of <0.05 were retained in multivariate models. α1AG, α1 acid glycoprotein; α1AT, α1-antitrypsin; bOPV, bivalent oral poliovirus vaccine; GMC, geometric mean concentration; IND, India; IPV, inactivated poliovirus vaccine; LRT, likelihood ratio test for fit of model with vs without polio vaccine schedule; MLW, Malawi; MPO, myeloperoxidase; neo+, infected with rotavirus neonatally (defined by detection of rotavirus shedding in week of life 1 or baseline seropositivity); neo-, uninfected with rotavirus neonatally; ORV, oral rotavirus vaccine; RR, relative risk; RV, rotavirus; tOPV, trivalent oral poliovirus vaccine; * log-transformed to approximate normality in statistical models; † excluded to minimise multicollinearity; § +2 weeks in UK due to later vaccination schedule.

File Name: Supplementary Data 3

Description: **Cofactors Associated with Post-vaccination Rotavirus-specific IgA Concentration.** Linear regression was used to explore the relationship between each covariate and post-vaccination RV-IgA (log-transformed). Variables with a univariate p value of <0.05 were retained in multivariate models. α1AG, α1 acid glycoprotein; α1AT, α1-antitrypsin; bOPV, bivalent oral poliovirus vaccine; GMC, geometric mean concentration; IND, India; IPV, inactivated poliovirus vaccine; LRT, likelihood ratio test for fit of model with vs without polio vaccine schedule; MLW, Malawi; MPO, myeloperoxidase; neo+, infected with rotavirus neonatally (defined by detection of rotavirus shedding in week of life 1 or baseline seropositivity); neo-, uninfected with rotavirus neonatally; ORV, oral rotavirus vaccine; RV, rotavirus; tOPV, trivalent oral poliovirus vaccine; * log-transformed to approximate normality in statistical models; †excluded to minimise multicollinearity; § +2 weeks in UK due to later vaccination schedule.

File Name: Supplementary Data 4

Description: **Geographic Discrepancies in Genus Composition.** Data are presented for (A) Malawi vs India, (B) India vs UK, and (C) Malawi vs UK. Genera are included if they had an FDR-adjusted p value of <0.05 based on either two-sided Fisher's exact test (differences in prevalence) or Aldex2 with two-sided Wilcoxon rank sum test (differences in abundance). Genera were tested if they were present in at least 5% of samples in one of the cohorts being compared. FDR, false discovery rates.
