# Supplementary material for: Impact of maternal antibodies and microbiota development on the immunogenicity of oral rotavirus vaccine in African, Indian, and European infants
Source: Nat Commun. 2021 Dec 15;12:7288. doi: 10.1038/s41467-021-27074-1 (PMC8674366; doi:10.1038/s41467-021-27074-1)
Supplement: Supplementary file 8 — Reporting Summary [file 41467_2021_27074_MOESM8_ESM.pdf]

nature portfolio

Corresponding author(s): Edward Parker

Last updated by author(s): Oct 27, 2021

# Reporting Summary

Nature Portfolio wishes to improve the reproducibility of the work that we publish. This form provides structure for consistency and transparency in reporting. For further information on Nature Portfolio policies, see our Editorial Policies and the Editorial Policy Checklist.

## Statistics

For all statistical analyses, confirm that the following items are present in the figure legend, table legend, main text, or Methods section.

| n/a | Confirmed | |
|---|---|---|
| ☐ | ☒ | The exact sample size ($n$) for each experimental group/condition, given as a discrete number and unit of measurement |
| ☐ | ☒ | A statement on whether measurements were taken from distinct samples or whether the same sample was measured repeatedly |
| ☐ | ☒ | The statistical test(s) used AND whether they are one- or two-sided<br>*Only common tests should be described solely by name; describe more complex techniques in the Methods section.* |
| ☐ | ☒ | A description of all covariates tested |
| ☐ | ☒ | A description of any assumptions or corrections, such as tests of normality and adjustment for multiple comparisons |
| ☐ | ☒ | A full description of the statistical parameters including central tendency (e.g. means) or other basic estimates (e.g. regression coefficient) AND variation (e.g. standard deviation) or associated estimates of uncertainty (e.g. confidence intervals) |
| ☐ | ☒ | For null hypothesis testing, the test statistic (e.g. $F$, $t$, $r$) with confidence intervals, effect sizes, degrees of freedom and $P$ value noted<br>*Give P values as exact values whenever suitable.* |
| ☒ | ☐ | For Bayesian analysis, information on the choice of priors and Markov chain Monte Carlo settings |
| ☒ | ☐ | For hierarchical and complex designs, identification of the appropriate level for tests and full reporting of outcomes |
| ☐ | ☒ | Estimates of effect sizes (e.g. Cohen's $d$, Pearson's $r$), indicating how they were calculated |

*Our web collection on statistics for biologists contains articles on many of the points above.*

## Software and code

Policy information about availability of computer code

| | |
|---|---|
| Data collection | Sample processing and data acquisition were performed using the following instruments and associated software:<br>(i) Real-time PCR for rotavirus shedding: QuantStudio 7K/12K Flex (ThermoFisher) in India, 7500 Fast Real-Time PCR System (Applied Biosystems) in Malawi, and CFX qPCR Instrument (Bio-Rad) in the UK;<br>(ii) Real-time PCR for 16S amplicon pool quantification: Light Cycler LC480II (Roche);<br>(iii) Rotavirus antibody ELISAs: EON Universal Microplate Reader with ELX405R washer (BioTek);<br>(iv) Enteropathy marker ELISAs: EON Universal Microplate Reader with ELX405R washer (BioTek) in India, EZ Read 400 Microplate Reader with Asys Atlantis Microplate Washer (Biochrom) in Malawi, and Multiskan Spectrum (ThermoFisher) in the UK;<br>(v) DNA quantification: Quanti-it (ThermoFisher) and Qubit 3.0 (ThermoFisher);<br>(vi) Amplicon fragment analysis: 5300 Fragment Analyzer System (Agilent) for assessing individual samples, Bioanalyzer (Agilent) for assessing amplicon pools, and TapeStation (Agilent) for assessing individual samples in validation subset;<br>(vii) Amplicon pooling: mosquito X1 (TTP Labtech);<br>(viii) Size selection of amplicon pool: Pippin Prep (Sage Science) with 1.5% Agarose Gel Cassette (Labtech);<br>(ix) 16S rRNA amplicon sequencing: Illumina HiSeq2500 (v2 chemistry, 600 cycles in rapid run mode) and Illumina MiSeq (v3 chemistry, 600 cycles). |
| Data analysis | Cutadapt version 1.18, QIIME 2 (version 2018.11 with associated DADA2 plugin), R version 3.6.1, and R packages including decontam (version 1.6) and dada2 (version 1.14.1). Analysis code generated during this study are available on Github (https://github.com/eparker12/RoVI; DOI: 10.5281/zenodo.5528337). |

For manuscripts utilizing custom algorithms or software that are central to the research but not yet described in published literature, software must be made available to editors and reviewers. We strongly encourage code deposition in a community repository (e.g. GitHub). See the Nature Portfolio guidelines for submitting code & software for further information.

## Data

Policy information about availability of data

All manuscripts must include a data availability statement. This statement should provide the following information, where applicable:

- Accession codes, unique identifiers, or web links for publicly available datasets
- A description of any restrictions on data availability
- For clinical datasets or third party data, please ensure that the statement adheres to our policy

The raw sequence data generated during this study have been deposited in the European Nucleotide Archive under accession code PRJEB38948. Processed data and analysis code are available on Github (https://github.com/eparker12/RoVI; DOI: 10.5281/zenodo.5528337)). Taxonomy assignment was performed using the Silva rRNA database (version 132; DOI 10.5281/zenodo.1172782).

# Field-specific reporting

Please select the one below that is the best fit for your research. If you are not sure, read the appropriate sections before making your selection.

☒ Life sciences ☐ Behavioural & social sciences ☐ Ecological, evolutionary & environmental sciences

For a reference copy of the document with all sections, see nature.com/documents/nr-reporting-summary-flat.pdf

# Life sciences study design

All studies must disclose on these points even when the disclosure is negative.

| | |
|---|---|
| Sample size | As reported in the published protocol (Sindhu et al, BMJ Open 2017;7), we calculated that a sample size of 150 infants in India and Malawi would provide 80% power to detect a two-fold higher mean concentration of RV-IgG in infants who fail to seroconvert compared with those who seroconvert (assuming 40% seroconversion in each), while a sample size of 50 in the UK would provide 79% power to detect significant differences in RV-IgA by seroconversion status across the study sites (assuming 95% seroconversion in the UK). These sample sizes would also provide 95% power to detect significant differences in Shannon index according to seroconversion status in India and Malawi. The sample size in India (n = 307) exceeded these targets owing to the high recruitment rates in this cohort and the decision to merge the inactivated poliovirus vaccine and oral poliovirus vaccine arms in the final analysis. On the other hand, owing to challenges in recruitment and sample collection over the course of the study, the final samples size in Malawi (n = 119) fell short in these estimates. Since RV-IgG data were not available for the UK and Malawi, our final analyses of maternal antibodies across cohorts focused on RV-IgA. |
| Data exclusions | We enrolled 684 mother–infant dyads (395 in India, 187 in Malawi, and 82 in the UK). A total of 484 dyads (307 in India, 119 in Malawi, and 60 in the UK) met the primary endpoint for inclusion in the analysis (measurement of seroconversion or dose 1 shedding). Missing data (e.g. owing to incomplete sample availability or assay failure) were excluded on a case by case basis. The number of samples included in each analysis are specified throughout the manuscript text, tables, and figures. |
| Replication | To validate the robustness of the microbiota sequencing protocol, 270 faecal DNA samples (90 each from India, Malawi, and the UK) were shipped to a separate facility for independent 16S rRNA gene amplification and sequencing. Validation samples were randomised across three plates that were sequenced across two Illumina MiSeq runs (v3 chemistry). Alpha diversity, beta diversity, and genus relative abundances for paired sampled showed high consistency across sequencing facilities (e.g. R-squared value of 0.985 for Shannon index based on linear regression). |
| Randomization | Not applicable. This was an observational cohort study in which infants received routine vaccines according to the national immunisation schedule. |
| Blinding | Not applicable. This was an observational cohort study in which infants received routine vaccines according to the national immunisation schedule. |

# Reporting for specific materials, systems and methods

We require information from authors about some types of materials, experimental systems and methods used in many studies. Here, indicate whether each material, system or method listed is relevant to your study. If you are not sure if a list item applies to your research, read the appropriate section before selecting a response.

## Materials & experimental systems

| n/a | Involved in the study | |
|---|---|---|
| | x | Antibodies |
| x | | Eukaryotic cell lines |
| x | | Palaeontology and archaeology |
| x | | Animals and other organisms |
| | x | Human research participants |
| x | | Clinical data |
| x | | Dual use research of concern |

## Methods

| n/a | Involved in the study | |
|---|---|---|
| x | | ChIP-seq |
| x | | Flow cytometry |
| x | | MRI-based neuroimaging |

# Antibodies

Antibodies used

Rabbit rotavirus hyperimmune serum (CMC Vellore, in house, at a dilution of 1 in 1500 for IgA and 1 in 1000 for IgG), biotinylated rabbit anti-human IgA (Jackson ImmunoResearch Laboratories, CAT# 309-065-011, at a dilution of 1 in 3000), and biotinylated rabbit anti-human IgG (Vector Laboratories, CAT#BA-3080, at a dilution of 1 in 800). Antibodies were used alongside purified cell culture lysates (WC3, at a dilution of 1in 5 for IgA and 1 in 4 for IgG) or mock-infected MA104 cells (at a dilution of 1 in 5 for IgA and 1 in 4 for IgG).

Validation

Capture antibody (rabbit rotavirus hyperimmune serum):
Antibodies used in the rotavirus IgA and IgG assays were validated following in-house SOPs at the Christian Medical College in Vellore, India. The polyclonal antibody against rotavirus was raised in rabbits. Serial dilution experiments were performed to fix the dilution. All dilution experiments were performed as per the Protocols for Determination of Limits of Quantitation; Approved Guidelines' (Clinical and Laboratory Standards Institute, Volume 24 Number 34) and the ISO 15189 Medical Laboratories-Requirements for Quality and Competence, and are available on file within the laboratory

Biotinylated rabbit anti-human IgA and IgG:
Commercial secondary antibodies were reconstituted as per the manufacturers' instructions (https://www.jacksonimmuno.com/lots/000000095822 for IgA and https://vectorlabs.com/biotinylated-goat-anti-human-igg-antibody-gamma-chain-specific.html#biozbadges for IgA). Checkerboard and dilution experiments were performed to obtain the optimal signal to noise ratio as per the Protocols for Determination of Limits of Quantitation; Approved Guidelines' (Clinical and Laboratory Standards Institute, Volume 24 Number 34) and the ISO 15189 Medical Laboratories-Requirements for Quality and Competence, and are available on file within the laboratory.

## Human research participants

Policy information about studies involving human research participants

| | |
|---|---|
| Population characteristics | Pregnant women were enrolled during their third trimester at study sites in Vellore (India), Blantyre (Malawi), and Liverpool (UK). The mean maternal age was 23.5 in India, 26.2 in Malawi, and 34.6 in the UK. Following birth, infants were followed up until 4 weeks after their second dose of oral rotavirus vaccine (week of life 14 in India/Malawi; week of life 16 in the UK). Among the infants included in the final analysis population, 152/307 (50%), 60/119 (50%), and 24/60 (40%) were female in India, Malawi, and the UK, respectively. The mean birthweight was 2.96 kg in India, 3.02 kg in Malawi, and 3.68 kg in the UK. Exclusion criteria included: congenital immune deficiency; chronic renal or liver failure; other chronic illnesses which may affect immune function; non-singleton pregnancy; low birthweight or pre-term birth (<34 weeks gestation); congenital anomalies and other neonatal complications requiring prolonged hospitalisation; and delivery by elective caesarean (UK only). In India, 70/307 (23%) infants were born by caesarean section. |
| Recruitment | At all sites, participants were recruited after receiving information on the study during routine antenatal visits. Study posters and leaflets were provided at antenatal clinics.<br><br>In the UK, study information leaflets were mailed to all pregnant women registered for an antenatal visit at the Liverpool Women's Hospital. Women were given the opportunity to contact study midwives if they wished to discuss the study further by telephone or during an upcoming clinic visit.<br><br>In Malawi, study personnel did regular oral presentations describing the study to pregnant women and their families waiting for antenatal consultations or Expanded Program on Immunization clinics at health centres.<br><br>In India, field research assistants conducted an awareness programme in the communities regarding the study by house-to-house visits. An enumeration of all pregnant women in the study area was simultaneously carried out, with the collection of information on gestational age, expected date of delivery, and the planned place of delivery. Pregnant women in their third trimester were offered the opportunity to visit the study clinic to learn more about the study and decide on their potential participation.<br><br>Willingness to volunteer for a longitudinal research study may have resulted in recruitment biases in each of the three cohorts. However, several of the primary objectives compared endpoints between rather than within the cohorts, and would thus be unaffected by these local recruitment biases. Moreover, oral rotavirus vaccine response followed the expected geographic gradient across the three cohorts. We therefore consider the mother–infant dyads included in this study to be a valid reflection of their local populations. |
| Ethics oversight | The study was approved by the Institutional Review Board at the Christian Medical College (CMC) in Vellore, the College of Medicine Research and Ethics Committee in Blantyre, and the North West – Liverpool East Research Ethics Committee in Liverpool. |

Note that full information on the approval of the study protocol must also be provided in the manuscript.
